# Spinal cord morphology predicts curve progression in adolescent idiopathic scoliosis treated with bracing? A prospective cohort study with magnetic resonance imaging

**DOI:** 10.1186/1748-7161-10-S1-O49

**Published:** 2015-01-19

**Authors:** Winnie CW Chu, Min Deng, Steve CN Hui, Fiona WP Yu, Tsz-ping Lam, Yong Qiu, Jack CY Cheng

**Affiliations:** 1 Department of Imaging and Interventional Radiology, The Chinese University of Hong Kong, Hong Kong; 2 Department of Orthopaedics and Traumatology, The Chinese University of Hong Kong, Hong Kong; 3 Spine Surgery, The Affiliated Drum Tower Hospital of Nanjing University Medical School, Nanjing, China; 4 Joint Scoliosis Research Center of the Chinese University of Hong Kong and Nanjing University, China

## Objective

The purpose of this study was to identify the neural morphological predictors measured by magnetic resonance imaging (MRI) for curve progression in adolescent idiopathic scoliosis (AIS) patients treated with bracing.

## Material and methods

A total of 66 AIS girls with right-sided thoracic/thoracolumbar curves (mean age, 12.6 years; range, 10 -14 years) prescribed for bracing treatment were enrolled. Longitudinal follow-up at 6-month intervals was made beyond skeletal maturity. Demographic and anthropometric parameters, curve magnitude, menarche status, and Risser sign were assessed at each clinic visit. Bone mineral density (BMD) at femoral neck and MRI measurements including ratio of spinal cord to vertebral column length, ratio of anteroposterior (AP) and transverse (TS) diameter of cord, lateral cord space (LCS) ratio, cerebellar tonsil level, conus medullaris position were obtained at baseline. AIS girls were assigned into three groups according to bracing outcome: (A) Non-Progression (curvature increase<=5 degree); (B) Progression (curvature increase>=6 degree); (C) Progression with surgery indication (Cobb angle>=50 degree after skeletal maturity despite bracing). The predictors for curve progression were evaluated using univariate analysis and multivariate ordinal regression model.

## Results

The average duration of follow-up was 3.4 years (range: 2.0-5.6 years). Of 66 AIS girls, there were 25 girls (38%) in group A, 21 girls (32%) in group B and 20 girls (30%) in group C, respectively. No significant intergroup differences were found in spinal cord length, cerebellar tonsil level and conus position (all P>0.05). Group C had significantly longer vertebral column length (P=0.026), smaller cord-vertebral length ratio (P=0.012), and higher AP/TS cord ratio (P=0.015) as compared to group A, while LCS ratio in group C was significantly increased when compared with both group A (P=0.005) and group B (P=0.016). In final regression model, four significant independent predictors including LCS ratio (Odds Ratio (OR): 3.053 [95% CI: 1.117-8.343, P=0.030]), initial curve magnitude (OR: 1.054 [95% CI: 1.004-1.108, P=0.036]), menarche age (OR: 2.126 [95% CI: 1.143-3.952, P=0.017]), BMD (OR: 3.009 [95% CI: 1.175 7.705, P=0.022]), and one marginally significant predictor e.g. AP/TS cord ratio (OR: 1.841[95% CI: 0.955-3.55, P=0.068]) were obtained.

## Conclusions

On baseline MRI measurement, LCS ratio is identified as a new significant independent predictor for curve progression in AIS while AP/TS cord ratio is suggested as a potential predictor which requires further validations. The above MRI parameters can be taken into accounts for prognostication of bracing outcome.

